# The Role of Carrageenan in Inflammatory Bowel Diseases and Allergic Reactions: Where Do We Stand?

**DOI:** 10.3390/nu13103402

**Published:** 2021-09-27

**Authors:** Barbara Borsani, Raffaella De Santis, Veronica Perico, Francesca Penagini, Erica Pendezza, Dario Dilillo, Alessandra Bosetti, Gian Vincenzo Zuccotti, Enza D’Auria

**Affiliations:** Department of Pediatrics, Vittore Buzzi Children’s Hospital, University of Milan, 20122 Milan, Italy; raffaella.desantis@unimi.it (R.D.S.); veronicaperico.94@gmail.com (V.P.); francesca.penagini@asst-fbf-sacco.it (F.P.); erica.pendezza@unimi.it (E.P.); dario.dilillo@asst-fbf-sacco.it (D.D.); alessandra.bosetti@asst-fbf-sacco.it (A.B.); gianvincenzo.zuccotti@unimi.it (G.V.Z.); enza.dauria@unimi.it (E.D.)

**Keywords:** carrageenan, processed foods, dietary pattern, inflammation, gut microbiota, inflammatory bowel diseases, allergic reactions

## Abstract

Carrageenan (CGN) is a high molecular weight polysaccharide extracted from red seaweeds, composed of D-galactose residues linked in β-1,4 and α-1,3 galactose-galactose bond, widely used as a food additive in processed foods for its properties as a thickener, gelling agent, emulsifier, and stabilizer. In recent years, with the spread of the Western diet (WD), its consumption has increased. Nonetheless, there is a debate on its safety. CGN is extensively used as an inflammatory and adjuvant agent in vitro and in animal experimental models for the investigation of immune processes or to assess the activity of anti-inflammatory drugs. CGN can activate the innate immune pathways of inflammation, alter the gut microbiota composition and the thickness of the mucus barrier. Clinical evidence suggests that CGN is involved in the pathogenesis and clinical management of inflammatory bowel diseases (IBD), indeed food-exclusion diets can be an effective therapy for disease remission. Moreover, specific IgE to the oligosaccharide α-Gal has been associated with allergic reactions commonly referred to as the “α-Gal syndrome”. This review aims to discuss the role of carrageenan in inflammatory bowel diseases and allergic reactions following the current evidence. Furthermore, as no definitive data are available on the safety and the effects of CGN, we suggest gaps to be filled and advise to limit the human exposure to CGN by reducing the consumption of ultra-processed foods.

## 1. Introduction

Over the last decades, intestinal and systemic immune-inflammatory disorders have increased, due to several factors including dietary habits. In the Western Diet food additives are widely used to preserve food quality.

Our interest in deepening the particular role of carrageenan (CGN) arises from many reasons.

In recent years, there has been a significant increase in the use of carrageenan as a food additive in the Western diet (WD) [1]. Although there are still limited data on the daily amount of food additives in the diet of paediatric population with IBD, carrageenan is one of the most frequently consumed ones, with mean exposures per day equal to 0.58 ± 0.63 in a paediatric group with Crohn’s disease [2]. Lastly, but not least, although evidence is still limited, a possible role of a diet low in carrageenan to prevent an earlier relapse of disease in IBD has been hypothesized [3].

The present review focuses on the role of CGN in inflammatory bowel diseases and allergic reactions. Other food additives potentially related to IBD are listed in Table 1.

Carrageenan is a family of high molecular weight, sulphated polysaccharides [1], composed of D-galactose residues linked in alternating β-1,4 and α-1,3 galactose-galactose bond [4]. It is isolated from the cell walls of red seaweeds [5], especially from *Chrondrus crispus*, *Gigartinastellata* and *Euchema* species of the class Rhodophyceae [6].

The chemical name of carrageenan is sulfate esters of polygalactose, and it appears as an odourless yellowish to colourless, coarse to fine powder [7].

**Figure 1:**
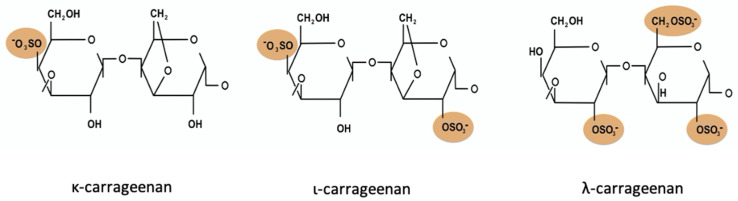
κ- ι- and λ-carrageenan primary structure.

CGN has no nutritive value, and it is widely used as a food additive (E-407) in processed food for its properties as a thickener, gelling agent, emulsifier and stabilizer [6]. CGN is also used in pet food, cosmetics, textiles formulations and pharmaceutical industries [8]. The major types, commonly used in food, are ι (iota), κ (kappa) and λ (lambda), which differ in the repetition of the unit sulphation (1, 2 or 3 sulphate group) [9] (Figure 1).

CGNs can be classified by their molecular mass (100–1000 kDa) through exclusion chromatography (SEC), by the degree of sulphation and by the position/distribution of the sulphate group.

A method to determine the extent of sulphation was described by Food and Agriculture Organization of the United States (FAO)/World Health Organization (WHO) Joint Expert Committee on Food Additives (JEFCA). The method provides an acid hydrolysis of the sulphate and subsequent precipitation of the sulphate ions as barium sulphate, measured by weighing or turbidimetry [5].

Many processed foods, especially reduced-fat or non-fat food products, such as ice cream, soymilk, yogurt, chocolate milk, salad dressing, beer, deli meats, and nutritional supplements, contain CGN (Table 2). In processed poultry, ham, and red meat products, carrageenan increases the yield by trapping water in the meat and binds meat juices to prevent their loss. It is also used as a browning inhibitor for fresh fruit processing control [10].

In the United States, in the 1970s, a daily intake of carrageenan in food of about 100 mg/day was estimated in adults. CGN’s use as a food additive has substantially increased over the last years especially with the spread of the Western diet [1].

In the typical Western diet average daily intake of CGN was estimated to be 250 mg/day [11]. More recently, a daily intake of 18–40 mg/kg/day was assessed by food industries reports [4,12].

JECFA in 1984 assigned an Acceptable Daily Intake (ADI) of ‘not specified’ and recognized a limit for carrageenan of 5 mg/kg. These indications have been reconfirmed at the subsequent meetings [13].

In 2015, the JECFA stated the safety of CGN, permitting its use also in infant formula [5].

In the United States, CGN has been used for decades as a stabilizer in infant formula. It binds strongly with the casein proteins to build a structure, provides the desired mouth feel, and stabilizes the fat emulsion. Regular milk and soy-based liquid infant formula can contain a maximum of 0.03 g/100 mL of CGN, while a maximum of 0.1 g/100 mL is permitted in hydrolyzed protein or amino-acid-based liquid infant formula [13,14].

Several in vitro studies have revealed that CGN is able to induce inflammation [15,16,17], triggering innate immune pathways of inflammation, involving the canonical and noncanonical pathways of NF-kB activation with a central role in transcriptional activation of the IL8 gene.

For this pro-inflammatory activity, CGN has been widely used as an inflammatory and adjuvant agent in experimental models (animal and cell-based experiments) for investigation of immune processes or to assess the activity of anti-inflammatory drugs [13,18].

For example, intramuscular injection of 10 mg of λ-carrageenan in rats caused chronic inflammation. This model of inflammation has been used to investigate the anti-inflammatory and immunomodulatory properties of mesenchymal stem cells (MSCs) [19].

Despite the CGN use as food additive, there is a debate on its safety, in particular regarding poligeenan, also called degraded CGN (dCGN), a fraction of CGN with lower molecular weight (average molecular weight < 20000 Da) that is formed through acid hydrolysis at pH 0.9–1.3 and heating (>80 °C) of CGN for several hours [7].

The fate of carrageenan in vivo is still matter of debate: previous evidences support the hypothesis that the formation of poligeenan in the human gastrointestinal (GI) tract does not occur due to the lack of the pH and temperature conditions needed to produce these molecules. Moreover, excretion studies indicate that 98–100% of the ingested CGN is excreted unchanged without significant degradation [12,14].

However, it is probable that some dCGNs are produced by acid hydrolysis during gastric digestion. Latest investigations observed that during gastrointestinal metabolism, CGN is degraded in smaller molecular weight components determining the activation of pro-inflammatory cytokines [5,18].

In animal models, poligeenan has been used widely to induce ulcerations, neoplasms and colorectal tumours. For this reason, dCGN was classified as possible human carcinogen (Group 2B) by IARC (International Agency for Research on Cancer) in 1982 [9,20].

Individual reaction to ingested carrageenan depends on different conditions in the gastrointestinal tract, such as gastric acidity, mucosal integrity, and previous damage. CGN can be desulfate from some colonic bacteria that leave only a degradable galactan polymer backbone. Bacteria can ferment these galactan polymers to produce short-chain fatty acids with the removed sulfate groups reduced by sulfate reducing bacteria (SRB) to sulfides. High levels of sulfides in the gut have been implicated in initiation of acute and chronic inflammatory diseases of the large bowel [10].

## 2. Dietary Pattern and Inflammation

Chronic inflammatory state is a condition characterized by a progressive inflammatory response and tissue destruction involved, underlying many pathological conditions, including those primarily involving the gastrointestinal tract, like inflammatory bowel diseases [19,21].

Diet has an important role among the modifiable and nonmodifiable risk factors for inducing systemic low-grade inflammation because it can have both a direct effect, by supplying nutrients and bioactive compounds that modulate inflammatory response, and indirect effects, by affecting body weight gain [22].

Studies on plant-based diets, like the Mediterranean diet, reported a reduction in the oxidative stress concentration and pro-inflammatory biomarkers and an increase in the antioxidant concentration and anti-inflammatory biomarkers. On the contrary, studies on Western and fast-food diets reported higher levels of oxidative stress and inflammation biomarkers [23].

The Mediterranean diet is characterized by a high intake of fruit, vegetables, legumes, cereals, seeds and nuts, low-to-moderate intake of dairy products, poultry, fish and wine and low intakes of red meat and eggs, with olive oil used as a main source of fat. This pattern provides an important number of bioactive compounds, such as polyphenols and dietary fibers. Observational studies suggest an inverse association between dietary polyphenols intake and the risk of cardiovascular disease, inflammatory and metabolic disorders and some types of cancer. This finding may be explained by the fact that they participate in several cell signaling pathways, in the downregulation of the transcription of pro-inflammatory cytokines, and in the reactive oxygen species production.

Furthermore, fibers can lower the glycaemic index and glycaemic load of meals while playing a role in gut microbiota modulation and gut permeability regulation.

This dietary pattern is inversely associated with plasma C-reactive protein (CRP) levels, an acute-phase protein synthesized in hepatocytes under stimulation from interleukin (IL)-6 and positively associated with plasma adiponectin levels (an anti-inflammatory adipocytokine), as outlined by a recent systematic review [22].

The Western diet is characterized by high consumption of calorically rich, processed foods, ‘‘fast food,’’ convenience products, snacks, and sugary soft drinks, with a very low consumption of fibers, vitamins, and minerals [24].

Ultra-processed foods are one of the main characteristics of the WD, and the environment produced in the gut by ultra-processed foods could be a soil for microbes that foster different forms of inflammation-related diseases, due to higher levels of endotoxin-producing bacteria in the intestinal tracts. An animal study demonstrated that a lack of fermentable fibers leads to changes in the microbiota resulting in intestinal inflammation [25].

A concomitant rise in Western-diet-associated diseases has occurred with a rise in mostly preventable diseases linked to a chronic inflammatory state, such as inflammatory bowel diseases and colorectal malignancy [18,19].

Long-term consumption of WD can influence physiology and health by promoting weight gain, pathological changes in lipids and energy metabolism, as well as activation of the immune system [24]. WD is also associated with a reduced gut microbial diversity (dysbiosis), which in turn may result in increased susceptibility to IBD and other common chronic diseases such as obesity and diabetes, by triggering and sustaining inflammation [26,27].

## 3. Gut Microbiota and Inflammation

Dietary behavior can result in some species dominating the gut more than others.

It has been shown that people who consume more meat in their diets have significantly different gut microbiomes, with a prevalence of *Bacteroides* species, to those with plant-based diets, with a prevalence of *Prevotella* species.

The Western diet, rich in saturated fats and low in unsaturated fats, is found to be positively associated with anaerobic micro-organisms and specific genera including *Bacteroides* and *Bilophila*.

In addition, animal studies have noted that additives commonly found in the Western diet have been associated with altered microbiota composition and its inflammatory potential [28].

The additives commonly used in processed food are frequently non-absorbed and they can directly interact with the microbiota.

Studies conducted in mice demonstrated that food additives can promote chronic intestinal inflammation and, for example, carrageenan induces intestinal inflammation in rodents and significantly alters microbiota composition [29,30].

Naimi et al. conducted an experiment on the effect of dietary additives on microbiota composition. A fecal sample was collected from healthy individuals and maintained ex vivo in a MiniBioReactor Array model. The results showed a reduction in *Lactobacillales*, consequently to a significant decrease in the *Streptococcus* genus. *Bacteroides* was significantly enriched by kappa and lambda carrageenans while iota carrageenan decreased the relative abundance of *Faecalibacterium*, also known for its anti-inflammatory properties.

These dietary emulsifiers induce slow but persistent increase in the microbiota’s expression of these pro-inflammatory molecules [31].

## 4. In Vitro Evidence

**Figure 2:**
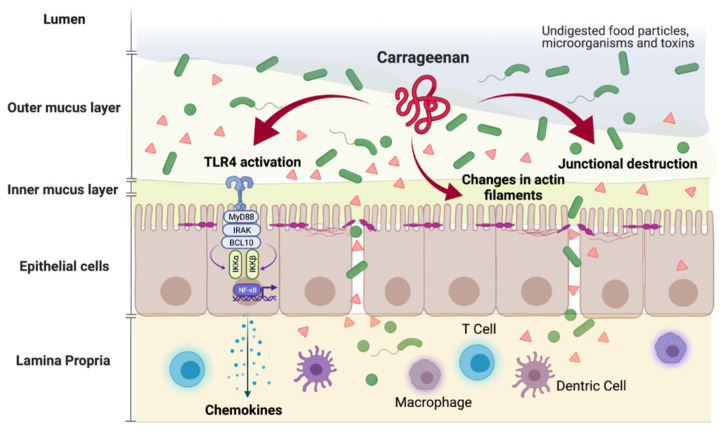
Carrageenan triggers the activation of the immune system and the development of inflammation (Made by © BioRender 2021).

Food additives, such as CGN, have been employed for a variety of functions but in vitro and animal studies have suggested the pro-inflammatory effect of several of them. Pre-clinical studies showed some of these additives to be linked with alterations in the intestinal microbiome, decreased thickness of the mucus barrier protecting the intestinal epithelium and intestinal inflammation (Figure 2) [28,31,32].

It has been suggested that CGN may act through several mechanisms: through the modifications of gastric proteolysis and affecting the gut epithelial structure and function.

Gastric proteolysis may be involved in the modulation of the immune system. This assumption is supported by the knowledge that most peptides bearing biological activity are released by enzymatic hydrolysis. For example, the proteolytic digestion of α-Lactoalbumin and β-Lactoglobulin (α-LA and β-LG) by endopeptidases product bioactive peptides with bactericidal properties, mainly against Gram-positive bacteria [33,34]. Accordingly, possible modifications in protein and peptide bioaccessibility, as a result of interference from ingested CGN, may favor gut microbiota changes leading to dysbiosis [9].

A recent study [9] used an in vitro human gastric digestion model and Caco-2 cell cultures (Human Colon adenocarcinoma Caco-2 Cells) to elucidate the possible mechanism of action of CGN on epithelial cell functions and its implications to the gastric degradation of food proteins. Electrostatic interactions between CGN and edible proteins are determined by the physicochemical differences between the various CGN types. This results in an alteration of the gastrointestinal degradation of proteins, and it diversely affects numerous structural and functional aspects of normal epithelial cell functions. In this study, three commercial carrageenans (ι, κ, λ) and food-grade whey protein isolate (milk, soy, egg) mixtures (CGN-WPI) have been used. The results found varying levels of interference between CGNs and gastric digestive proteolysis and a significant decrease in pepsin activity.

The different CGNs used in the food industry significantly vary in their ζ-potential depending on the range of pH tested and the CaCl_2_ concentrations arising from different degree of sulphation. Moreover, at typical luminal physiological conditions (5 mM CaCl_2_), the different CGN types vary in their ζ-potential, then CGN varies in charge and maintains this variability under conditions relevant to human digestion.

This evidence is supported by previous studies which showed that CGN is able to inhibit human gastric juices and pepsin compromising the digestive proteolysis [35].

Inhibition by degraded carrageenan is constant through the pH range 1.5–3.75, but inhibition by undegraded carrageenan decreases between pH 2.5 and 3.25. This inhibition is caused by substrate-inhibitor interaction. The differing natures of the substrate-inhibitor complexes formed by degraded and undegraded carrageenans influence the differences in degree of inhibition and the effect of pH [35]. In this study, CGN significantly (*p* < 0.05) interfered with pepsin activity, consequently the increased resistance to proteolysis may have an impact on bioactive peptides and allergens in the intestinal lumen. Moreover, the physiologically digested CGN (pdCGN) in the Caco-2 cell cultures affected the epithelial barrier function, with the redistribution in a dose-dependent manner, in the polarized epithelial cells to more basal parts compared with controls of the tight-junction protein Zonula Occludens-1 (ZO1). This relocation of ZO1 from the plasma membrane to internal compartments of the epithelial cells occurs in response to the exposure to pdCGN [9].

Furthermore, changes in cellular F-actin architecture (condensed actine filaments) increased monolayer permeability and contraction of apical actin ring may lead to the disruption of the intracellular junction between adjacent cells, thus impeding the barrier function these junctions provide. Leaky gut barriers cause an increase in intestinal permeability and a decrease in transepithelial electrical resistance (TEER), an index of confluence and integrity of epithelial monolayer cells, which in turn may favor translocation of luminal bacteria and subsequent inflammatory responses [36].

Additionally, pdCGNs induced inflammation by the activation of nuclear factor K (NF-kB) and IL8 production. The levels of CXCR1 receptor were significantly increased with λ and ι pdCGNs compared to the control group [9].

Previous in vitro studies carried out according to protocols with reduced bio-relevance indicate that low concentrations of undigested CGN cause activation of inflammatory pathways through the nuclear translocation of the NFkB transcription factor and the induction of IL8 [15,16,17].

Some studies have shown that one of the factors involved in the gut dysbiosis can be the increased proteolytic activity over saccharolytic activity in the colon [37,38].

In human colonic epithelial cells line, CGN induce inflammation triggering innate immune pathways of inflammation, involving the canonical and noncanonical pathways of NF-kB activation. The interaction of CGN with the toll-like receptor (TLR)-4 results in interaction of BCL10 with IKKy, the regulatory subunit of the IKK signalosome, leading to ubiquitination of IKKy, IKKβ phosphorylation of IkBa, and nuclear translocation of NF-kB RelA (p65) and p50. Otherwise, as in the noncanonical NF-κB activation pathway, phospho-BCL10 interacts with NIK which is phosphorylated, leading to the phosphorylation of IKKα and the nuclear translocation of RelB and p52. Lastly NF-κB plays a central role in transcriptional activation of the IL-8 gene. Furthermore, in addition to TLR4-BCL10 mediated pathways, CGN stimulates a reactive oxygen species (ROS) mediated pathway. In this cascade, phospo-Hsp27 interacts with the IKK signalosome, leading to the phosphorylation of IkBa and the nuclear translocation of NF-kB. A feedback loop is established to increase production of BCL 10 with NF-kB binding to the BCL10 promoter in order to maintain activity in the inflammatory process [15,17,39,40].

## 5. Animal Models Evidence

The effect of carrageenan as a pro-inflammatory substance has been observed at a cellular level by means of in vitro studies, but its safety in animal models is still highly controversial. Some studies on animal models have indicated that carrageenan intake (average daily intake between 1.7–41.7 mg/kg) encourages colonic inflammation, exhibiting development of inflammatory infiltrates and clinical evidence of colitis. Based on these findings, we can assume that carrageenan might be a “recessive inflammatory agent”, meaning that CGN might enhance the inflammatory response, acting in a synergic way, when the intestinal tract is compromised, for example during an acute inflammation [17,41].

The ability to activate the innate immune system is attributed to the distinctive chemical structure of CGN.

Carrageenan possesses the unusual α-1→3 galactosidic bond that is absent in humans and therefore is recognized as an immune epitope. The interaction between this immune epitope and the toll-like receptor 4 (TLR4) lead to the activation of the innate immune response.

Moreover, specific bacteria of the colonic microflora are able to produce galactosidases and carrageenases, with the subsequent production of dCGN and a greater interaction between alpha-gal epitope and TLR4.

The development of intestinal inflammation from exposure to CGN may vary from individual to individual or over time in the same individual due to the different composition of the microbiota [42].

Benard et al., in 2010, aimed to analyze the size-dependent effects of dCGN on colonic inflammation in vivo. Male rats were fed ad libitum with standard rodent laboratory chow. The rats were divided into groups of six animals each, and degraded ι-carrageenan was administrated in the drinking water. The first group received a 10 kDa dCGN, the second one received a 40 kDa dCGN and the third group was the control group that did not received degraded ι-carrageenan. After 55 days, the investigators observed a strong correlation between the severity of the inflammation and the dCGN molecular size. All rats, except the control group, developed diarrhea and the colon length decreased especially in the group who received the 40 kDa dCGN [30].

Undeniable evidences show that low molecular weight (Mw) CGN or degraded CGN may be the culprit of the adverse effects associated to CGN. Macrophage lysosomal disruption and subsequent epithelial ulcerations are caused by degraded CGN as well as the increases in TNFα and ICAM secretion in monocytes [18,30]. The hypothesis is that pdCGN containing raised levels of Mw CGN may have higher diffusion rate to the epithelium that may lead to greater exposure to the toxic low Mw CGN species [5]; however, these assumptions need to be confirmed.

There is a gap of extensive controlled human trials or epidemiological studies that refute or support the studies suggesting that CGN has the capacity to induce inflammation in vivo. The uncertainties about CGN effects are due to inconsistent reports and lack of methodological harmonization in CGN research.

Furthermore, there are insufficient data regarding the effects of physiologically digested CGN on gut and/or immune functions.

It is advisable to consider that results obtained from in vitro models cannot be generalized in animals or humans unless the cell-based model has been shown to possess the same functional mechanisms that exist in vivo [12].

Future research in more bio-relevant models is needed replicating the human physiological environment in which digested CGN interacts with the intestinal epithelium.

A proper model combined with chemical analysis of the digested CGN may help explain the questions concerning its implications to inflammatory pathway in vivo [5].

## 6. Carrageenan and Inflammatory Bowel Diseases

The intake of carrageenan in Western diet is sufficient to induce intestinal inflammation, as illustrated above. Gastric digestion or passage through intestinal bacterial flora could probably be responsible for the formation of small amounts of dCGN. Even in the general population, reports about dietary habits suggest that carrageenan consumption may be related to adverse effects. For instance, the Cornucopia Institute, a public interest group concerned with diet, recorded an improvement in gastrointestinal symptoms after the reduction of carrageenan content in diet, using online questionnaires [43].

Inflammatory damage associated with carrageenan in murine models shows a histopathological pathway similar to the one known in an inflammatory intestinal condition such as ulcerative colitis (UC). In summary, the carrageenan-activated cascade involves reactive oxygen species and the innate immune pathway including the toll-like receptor (TLR)-4 and B-cell leukemia/lymphoma (BCL)10 [14].

Given these assumptions, the following paragraphs summarize the scientific evidence currently available regarding the interaction of carrageenan with the main chronic inflammatory bowel diseases (IBD). Dietary factors have always been extensively examined for their possible role in pathogenesis of IBD [44]; on the other hand, clinical studies on the specific role of a single food additive, such as carrageenan, are still limited but the data already provided make further investigation desirable and useful.

The increased prevalence of IBD in the last years and its known association with Western diet [26,45] correlate with greater carrageenan consumption [46]. Different types of diets can contribute to the management of chronic inflammatory bowel diseases, and these diets turn out to be similar to the carrageenan-free diet because they advise to avoid dairy products and processed foods consumption. For instance, the Specific Carbohydrate Diet, avoiding consumption of grains, simple sugars (except for honey), dairy products or processed foods, improves intestinal inflammation acting on the balance of intestinal microbiota [47]. Similarly, the Mediterranean diet, advising to consume fish, fruits, vegetables, whole grains, legumes, olive oil and nuts, and to avoid dairy products (e.g., butter) and processed foods, can be useful in IBD management [48].

### 6.1. Carrageenan and Ulcerative Colitis

As mentioned above, carrageenan-induced inflammatory damage and histopathology of UC share a similar pathway.

In 2017, a randomized, double-blind, placebo-controlled, multicenter, clinical trial was conducted to study the effects of the no-carrageenan diet on UC disease activity [3]. In particular, all twelve participants followed a no-carrageenan diet, and they were randomized to either placebo capsules or carrageenan-containing capsules (the amount in capsules was less than the average daily intake in Western diet). They were interviewed every two weeks for a year or until a clinical relapse, defined as the increase of at least two points in the Simple Clinical Colitis Activity Index (SCCAI) and the need to intensify therapy. No clinical relapse was reported in patients receiving placebo capsules, while it was reported in three of the patients receiving carrageenan-containing capsules; also, only in this second group, increased levels of important inflammation markers such as interleukin 6 (IL6) and faecal calprotectin have been demonstrated. The role of carrageenan on clinical relapse can be explained through several mechanisms, such as interaction with the gut microbiome and direct activation of the inflammatory cascade in colon cells.

In conclusion, this study shows that carrageenan intake can be responsible for an earlier relapse in patients with ulcerative colitis in remission; for this reason, reducing carrageenan daily consumption can benefit these patients.

### 6.2. Carrageenan and Crohn’s Disease

It is now well known how diet can significantly influence the clinical course of paediatric Crohn’s disease (CD); indeed food-exclusion diets can be an effective therapy for inducing disease remission [49]. In particular, all these diets show as a common factor the avoidance of processed foods, that are widely consumed in the Western diet. Because food additives are associated, in pre-clinical studies, with intestinal microbiome alteration and inflammation, a study from 2018 tried to quantify food additive exposure in children with CD [31]. Results of the study found that in the CD population, there was a frequent exposure to xanthan gum, maltodextrin, soy lecithin and carrageenan. Other food additives that might be related to IBD in past studies are listed in Table 3.

Further clinical studies are therefore necessary to investigate the role of these food additives in the pathogenesis of CD.

## 7. Carrageenan and Allergic Reactions

A dated case report described a 26-year-old woman who experienced anaphylaxis during a barium enema study, conducted for gastrointestinal symptoms attributed to irritable bowel syndrome [57].

At first, the anaphylactic reaction was thought to be due to latex proteins because the literature reports some cases of anaphylaxis caused by latex present in the barium enema device.

However, no latex was found in the tip of the barium enema delivery system. In addition, skin prick tests (SPT) for latex resulted negative, while SPT with the barium enema solution used for the test resulted positive with a 10-mm wheal diameter.

Further SPT and search for specific IgE were performed, and both resulted positive for a sodium carrageenan solution. A more thorough history of the patient allowed to discover that her gastrointestinal symptoms worsened after the ingestion of some processed milk products (containing carrageenan), but not after plain milk consumption.

Due to the history of anaphylaxis, an oral food challenge with carrageenan was not performed, but allergy was diagnosed, and the patient was advised to exclude carrageenan-containing foods from her diet [57].

In 2018, Kular et al. published a case of carrageenan allergy in a 10-month-old infant, which is currently the only paediatric case reported in the literature.

The infant presented lip angioedema shortly after ingestion of icing from a fruit cake, with no other associated symptoms. He was known to tolerate the main “classic” food allergens, and other cake icings with no reactions. SPT for the cake icing resulted positive, but those for the main ingredients of the cake were negative. After the analysis of the ingredient list, carrageenan was found among the ingredients, SPT for carrageenan were performed and resulted positive.

So far, two cases of IgE-mediated adverse reactions after carrageenan ingestion have been described. They highlight the importance of recognizing carrageenan as a potential trigger for adverse reactions [58].

Specific IgE to the oligosaccharide α-Gal have been associated with episodes of delayed anaphylaxis to red meat in recent years. This alpha-gal-mediated response is commonly referred to as the “α-Gal syndrome” [59].

To date, mammalian meat products and tick bites have been identified as the main sources of exposure to the alpha-gal epitope [60].

The chemical structure of carrageenan intrinsically contains the alpha-gal epitope, to which anti-gal antibodies are formed. Considering that carrageenan has been increasingly incorporated into food products and processed foods, it may represent a potential source of exposure.

## 8. Conclusions

Carrageenan is widely used as a food additive (E-407) in processed food, and its use has increased in recent year. Despite the wide CGN use as food additive, its safety is still matter of debate.

In vitro and animal studies have suggested the pro-inflammatory effect of several food additives including CGN, but it is not feasible to attribute the same results obtained in in vitro and/or in animal models to humans, although the cell-based model has been shown to possess similar functional mechanisms that exist in vivo.

Furthermore, there is very scant available data on dietary intake of carrageenan and there is lack of evidence about the content of CGN in different dietary patterns and its effects on health.

To fill the gaps, future research should replicate human physiological conditions based on bio-relevant models and investigate the dietary intake of CGN.

Until no data on the safety and the effects of carrageenan are available, a reduction of human exposure to CGN limiting ultra-processed foods is advisable.

## Figures and Tables

**Table 1:** Mean exposures per day of carrageenan and other food additives in a population of 138 children with Crohn’s disease—Adapted from Dale Lee et al., 2018 [2].

Food Additive	Mean Exposure per Day
Xanthan Gum	0.96 ± 0.72
Maltodextrin	0.95 ± 0.77
Soy Lecithin	0.90 ± 0.74
Carrageenan	0.58 ± 0.63
Others (Carboxymethylcellulose, Polysorbate-80, Aluminosilicates, Titanium dioxide)	<0.1

**Table 2:** Products containing carrageenan—Adapted from Tarlo et al., 1995 [9].

Dairy Products	Ice Cream, chocolate Milk, Sorbets, Milk Desserts, Soymilk, Yogurt, Products Made from Fresh Cheese, Thickened and Sterilized Cream
Cured meat	Canned meat, pâtés, frozen food, glazed ham
Products with jelling agents	Jams and jellies, candied fruit, icing sugar
Powdered products	Instant drink, formulated baby milks, powdered milk desserts, hot milk pudding
Soup, sauces	Emulsified sauces (salad dressing, mayonnaise), gravies, soups

**Table 3:** Most common food additives consumed by a population of 138 children with Crohn’s disease [2].

Xanthan Gum	[50]
Maltodextrin	[51]
Soy Lecithin	[52]
Carboxymethylcellulose	[53]
Polysorbate-80	[54]
Aluminosilicates	[55]
Titanium dioxide	[56]
